# Dynamic selection of visible wavelengths using resonant TiO_2_ nanostructures

**DOI:** 10.1515/nanoph-2023-0057

**Published:** 2023-05-03

**Authors:** Han-Don Um, Deokjae Choi, Amit Solanki, Emerald Huang, Kwanyong Seo, Fawwaz Habbal

**Affiliations:** Department of Chemical Engineering, Kangwon National University, Chuncheon, Gangwon-do 24341, Republic of Korea; John A. Paulson School of Engineering and Applied Sciences, Harvard University, Cambridge, MA 02138, USA; School of Energy and Chemical Engineering, Ulsan National Institute of Science and Technology (UNIST), Ulsan 44919, Republic of Korea

**Keywords:** dielectric metasurface, hybridized modes, lattice resonance, magnetic and electric dipole resonances, titanium dioxide, tunable metasurface

## Abstract

All-dielectric nanoarrays have strong electromagnetic resonances with various interesting applications and are tuned by adjusting their geometrical parameters. However, their optical properties are permanently encoded during fabrication. This study presents robust dynamically tunable all-dielectric nanoresonators for controllable, reversible, and reproducible color filtering. Our design uses an array of TiO_2_ nanodiscs embedded in a transparent, stretchable polydimethylsiloxane (PDMS) membrane and exhibits a narrow spectral response due to Mie magnetic and electric dipole resonances hybridized with the TiO_2_ nanodiscs lattice modes. By mechanically stretching the PDMS membrane, the pitch of the TiO_2_ nanodiscs was increased and the spectral location of the resonances was altered. Additionally, an optically asymmetric structure was fabricated by partially embedding TiO_2_ nanodiscs in PDMS. Thus, the magnitude of the Rayleigh anomaly diffraction, which could interrupt the dipole resonances, was reduced. Our design has sharp, frequency-tunable resonances in the visible spectrum, and we demonstrated dynamic tunability by stretching the metasurfaces.

## 1 Introduction

Metasurfaces are optical materials with several innovative applications [1–8]. These materials have interesting light scattering properties, which enable the fabrication of small, lightweight optical devices, such as flat lenses [9], beam converters [10], deflectors [11], and holograms [12, 13]. Most dielectric metamaterials are arrays of subwavelength nanoresonators that strongly interact with the incident light [14–19], producing resonances that are utilized in optical devices. These strong optical resonances are induced by Mie resonances, and enhanced by the collective periodicity of the nanoresonators lattice [20]. The observed resonances are an outcome of the coupling between two fundamentally different resonances: the Mie resonances and the lattice resonance. The Mie scattering is related to the polarizability of the material and its composition, as well as the shape of the nanoresonators. The lattice resonance is related to the periodicity of the nanoresonator array. The hybridization of these two resonances represents coupling between the relatively broad Mie resonance of individual scatterers and discrete states associated with the light scattered by the periodic array [21–23]. This coupling is a function of several parameters such as the periodicity of the lattice structure and the optical properties of the surrounding medium.

The properties of the resonating elements and the lattice properties provide excellent opportunities for designing optical devices. Previous reports [24–30] have demonstrated that the optical responses are tunable by modulating the geometric parameters of the array. However, these electromagnetic resonances are then permanently encoded by the array’s geometry, which is formed during fabrication. Recently, attempts have been made to tune the metasurface resonances using various materials, such as liquid crystals [31–33], oxidized/reduced oxides [34], phase change materials [16], and solvents [35]. The dynamic modification of the metasurface performance using controllable, reversible, and reproducible methods is useful for various applications. Transparent and stretchable elastomers, such as polydimethylsiloxane (PDMS), have been successfully used as a substrate for tuning optical metasurfaces. By stretching the elastomer, the pitch (spacing) between the nanoresonators was changed to achieve different optical resonances [20].

This study presents a robust design for fabricating mechanically tunable all-dielectric nanoresonators for spectral filtering and other applications and shows conditions for observing both the electrical and magnetic resonances. A TiO_2_-based metasurface is fabricated using a procedure that is compatible with the industrially applicable complementary metal oxide semiconductor (CMOS) process [17].

Nanostructured TiO_2_ has a high index of refraction, *n*, larger than 2.5, and is suitable for imaging applications because it is transparent with insignificant optical losses in the visible spectrum [3]. A composite with a periodic square array of TiO_2_ nanodiscs embedded in a stretchable PDMS membrane is fabricated. By stretching the PDMS, the pitch between the TiO_2_ resonators changes, while the geometry of the hard TiO_2_ nanodiscs is maintained. Several TiO_2_ nanodiscs arrays are fabricated with dimensions appropriate to monitor the contributions of the electric and magnetic field components of the resonances and they exhibit sharp transmittance lines in the visible region of the electromagnetic spectrum.

It is recognized that the geometric parameters (diameter and height) of the array elements influence the resonances, and the collective behavior of the resonant elements affects the scattering properties and the corresponding resonances [1, 30]. Additionally, these collective electromagnetic resonances are sensitive to changes in the distance between the resonant nanostructures [36, 37]. This is a direct consequence of changes in the lattice resonances due to the change in the coupling between the adjacent resonant elements. When increasing the pitch, a longer wavelength of the light is needed to excite the same lattice resonances. Thus, spectral location of the resonances redshifts with increasing pitch. This dependence of the resonance wavelength on the pitch allows a dynamic tuning of the spectra.

Notably, there is another type of resonance that arises from the periodicity of the array. Any periodic scattering structure results in Rayleigh diffraction that may interact with the Mie lattice resonances, and even suppressing the lattice resonances. To clearly observe the dipole resonances, it is important to reduce the intensity of the Rayleigh diffraction by changing the index of the surrounding materials.

The optical characteristics of the lattice modes change with the pitch and the optical properties of the surrounding medium [38]. This was achieved by introducing optical asymmetry in the propagation direction of the incident light. With these changes, this study shows experimentally sharp optical resonances that are regulated by changing the pitch of the TiO_2_ nanoresonators and dynamically tuned in the visible spectrum. This type of spectral tuning is suitable for fabricating color filters that are dynamically selectable, which is useful for different imaging applications including multispectral spectroscopy.

## 2 Methods

### 2.1 Fabrication

Different PDMS-embedded TiO_2_ nanodisc arrays were prepared on quartz substrates. The quartz substrate was cleaned by sonication in acetone and isopropyl alcohol (IPA). The sacrificial germanium oxide layer was formed onto the quartz substrate by depositing a 100 nm-thick Ge layer using an e-beam evaporation system followed by annealing at 600 °C for 10 min under an O_2_ gas flow of 1000 sccm. This is a reliable method for fabricating a sacrificial germanium oxide layer. The TiO_2_ nanodisc arrays were formed using e-beam lithography followed by ALD deposition. To form the 275 nm thick electron-beam resist layer, a pristine electron-beam resist solution (ZEP520A, Zeon Corp.) was diluted with anisole solvent (99.7 %, Sigma-Aldrich) at a 1:2 volume ratio. The diluted e-beam resist solution was spin-coated onto the germanium oxide layer at a speed of 3000 rpm for 45 s. Subsequently, the substrate was annealed at 180 °C for 3 min. The desired hole pattern was written using e-beam lithography (ELS-125, Elionix) at a dose of 400 μC/cm^2^ and beam current of 1 nA, which produced the hole patterns over a 250 × 250 μm area. The hole diameter was 120 nm, and the pitch between the holes was 280 nm. The exposed e-beam resist was developed in anhydrous o-xylene (97 %, Sigma-Aldrich) for 45 s and then rinsed with IPA for 30 s. Subsequently, a 100 nm-thick TiO_2_ layer was deposited at 90 °C to fill the e-beam resist nanoholes. To remove the TiO_2_ layer that formed on top of the e-beam resist, the TiO_2_ surface was maintained at 20 °C and etched by the reactive-ion etching (RIE) process using BCl_3_ gas (40 sccm) and Ar gas (60 sccm), and source and stage powers of 500 W and 100 W, respectively. To prevent e-beam resist exposure, an O_2_ plasma treatment was performed under an O_2_ gas flow of 40 sccm at the room temperature, with a source power of 150 W and chamber pressure of 250 mTorr. To embed the TiO_2_ nanodisc array into PDMS, a mixed solution of PDMS base resin and curing agent (Sylgard 184, Dow Corning Co.) was spin-coated onto the TiO_2_ nanodisc array at a speed of 500 rpm for 3 min followed by annealing at 60 °C for 5 h. Subsequently, the cured PDMS with the TiO_2_ nanodisc array was immersed in water for 10 h to dissolve the germanium oxide layer, and finally the PDMS-embedded TiO_2_ nanodisc array was detached from the quartz substrate.

### 2.2 Optical characterization

Computer simulations of light propagating into the PDMS-embedded TiO_2_ nanodisc arrays were performed using a commercial software package (FDTD Solution, Lumerical Inc. and FDTD Band Solver, Optiwave systems Inc.) to obtain the transmission and reflection spectra. The refractive index of TiO_2_ used in the simulation was measured by ellipsometry (WVASE32, J.A. Woollam). For the optical simulation, a plane wave was injected onto the structure at a normal incidence with the electric field polarized along the *x*-axis. The *x* and *y* boundaries of the simulation domain were set with periodic boundary conditions. A perfectly matched layer at the *z* boundary of the domain was used, and simulations were performed with various unit cell sizes from 280 × 280 nm to 400 × 400 nm. Using a hyperspectral imaging microscope system (Horiba, XploRA), the reflection and transmission spectra of the samples were measured. To mechanically stretch the PDMS-embedded TiO_2_ nanodisc arrays, a homemade radial stretching system was designed. This radial stretching system was equipped with 6 arms that would serve to hold and stretch the sample. Each arm featured a peg that moves along a curved track on a rotating frame, allowing the arms to be pulled apart in a synchronized and controlled manner. The arms are guided within a fixed frame that is situated between two rotating frames. As a result, the sample dimensions can be increased from its original size by about 43 % (from 6 mm up to 8.5 mm).

## 3 Results and discussion

### 3.1 Key geometric parameter of TiO_2_ metasurface: pitch

**Figure 1:**
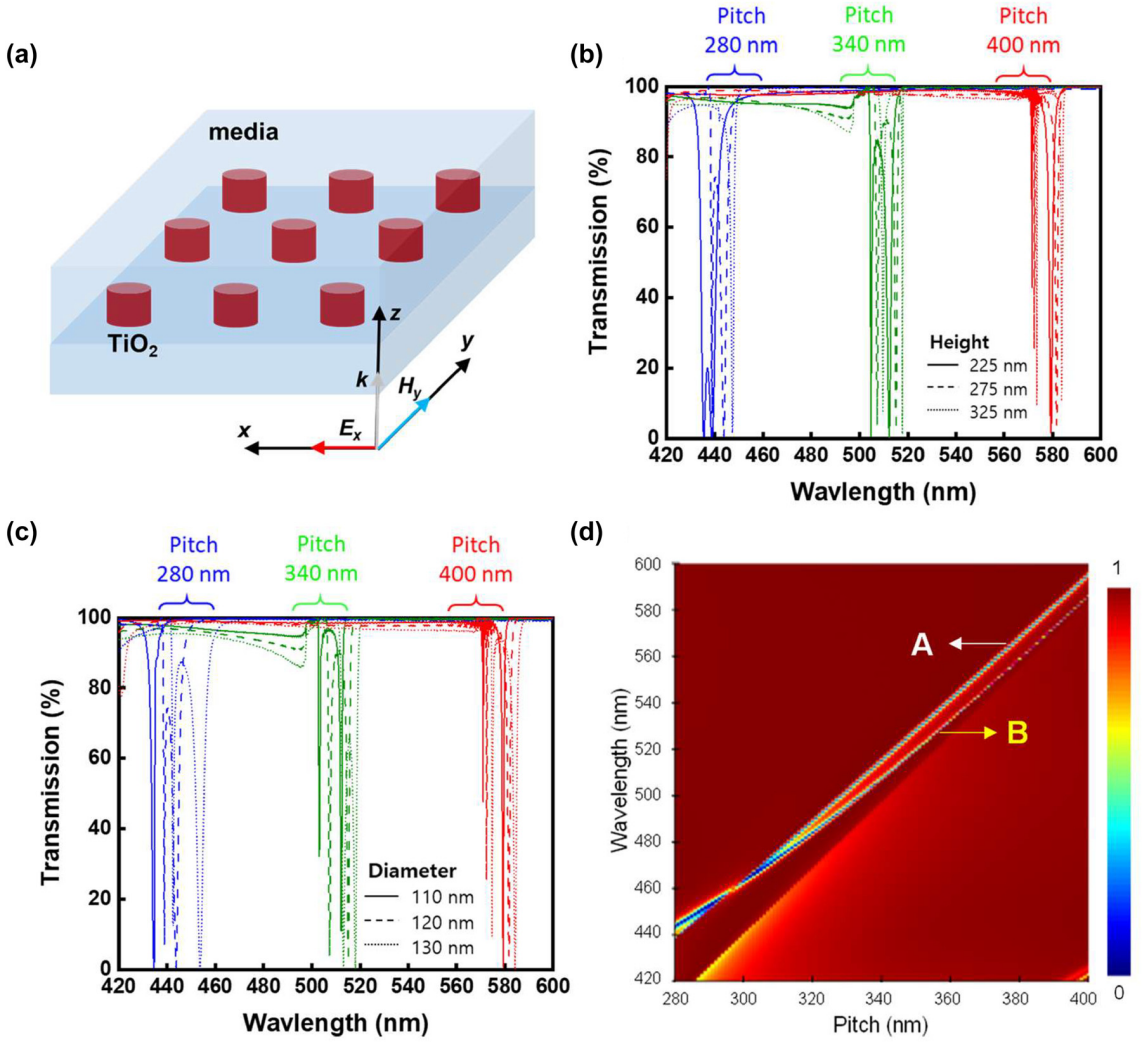
Simulation results of TiO_2_ nanodiscs completely embedded in uniform media with different indices. (a) Schematic of completely embedded TiO_2_ nanodiscs for the optical simulation. (b) Simulated transmittance spectra of TiO_2_ nanodiscs with different pitches and heights, at a fixed diameter of 120 nm. (c) Simulated transmittance spectra of TiO_2_ nanodiscs with different pitches and diameters, at a fixed height of 275 nm. (d) Transmittances versus pitch of the completely embedded TiO_2_ nanodiscs with a diameter of 120 nm and thickness of 275 nm.

To simulate the optical properties of the TiO_2_ metasurface, commercial software (Ansys Lumerical) was used to perform complete three-dimensional (3D) finite-difference time-domain (FDTD) analysis. First, an infinite array of TiO_2_ nanodiscs completely embedded in PDMS was considered, as shown in Figure 1(a), and the effects of changing the geometric parameters of the array were investigated. A unit cell of a single TiO_2_ nanodisc in the center with periodic boundary conditions in the *x* and *y* directions and a perfectly matched layer boundary in the *z* direction was used as the starting structure. A plane wave source with an electric field polarized along the *x*-axis and wave vector in the *z* direction was imposed onto the TiO_2_ nanodisc side and then propagated through PDMS. The reflectance and transmittance spectra were obtained by field monitors above and below the structure. The simulated optical response of the array was mapped by changing three geometrical parameters: nanodisc height (*H*), nanodisc diameter (*D*), and array pitch (*P*). Additionally, the refraction indices of the substrates and nanodiscs were used as simulation parameters. The refractive index of TiO_2_ used in the simulation was experimentally measured using a TiO_2_ thin film deposited by atomic layer deposition (ALD) (Supplementary Figure S1), which was also used to form the metamaterial TiO_2_ nanodiscs.

The transmission resonances were simulated for different heights at a fixed diameter. This was repeated for different pitches, as shown in Figure 1(b). The heights were 225, 275, and 325 nm, and pitches were 280, 340, and 400 nm, while the diameter was fixed at 120 nm. The transmittance spectra exhibited sharp decrease at particular spectral locations (dips), which did not significantly shift with changes in *H* in the range of 275 ± 50 nm. In contrast, the dips significantly shifted with increasing array pitch from 280 to 400 nm. Subsequently, the diameter was varied (*D* = 110, 120, and 130 nm) at a fixed height of 275 nm, and the simulations were repeated at different pitches. The transmittance spectra are shown in Figure 1(c). From Figure 1(b) and (c), the location of the spectral dips depended strongly on the array pitch and weakly on the diameter and height of the nanodiscs. To evaluate the spectral dependence on pitch, a simulation was performed using a fine pitch sweep with *P* = 280–500 nm for nanodiscs with a diameter of 120 nm and height of 275 nm, as shown in Figure 1(d). The spectral location of the transmission bands exhibited redshifts with increasing pitch, and crossover at *λ* ∼ 295 nm. These results are consistent with data in the literature [29]. The locations of the scattering modes are controlled by the size and refractive index of the nanostructures, i.e., the Mie scattering cross sections, and it is essential to have high index material to exhibit resonances in the visible wavelengths. On the level of the resonating elements, the electric dipoles scatter light symmetrically in the plane transverse to the dipole axis, and the resonant magnetic dipole results from a coupling of incoming light to the circular displacement currents of the electric field, due to the field penetration and phase retardation inside the nanodiscs. It is well known that the geometric parameters (diameter and height) of the elements of the array influence the resonances. The collective optical resonances are sensitive to the change in the distance between the resonant nanostructures [36, 37]. This is a direct consequence to changes in the coupling between adjacent elements. This electromagnetic coupling enhances with decreasing the pitch [37], and in a large array of resonators, the nearest neighbors provide the strongest contribution to the overall resonances. The electromagnetic coupling is sensitive to changes in pitch between the scattering centers, and reducing this coupling leads to changes in resonance intensities [24, 39]. When the spacing between the scattering elements increased, the coupling is significantly reduced and then diminished, and the resonance spectra disappeared (Figure 1(d)). One of the resonances diminished at pitch values higher than ∼385 nm, and further increasing the pitch above 545 nm weakened the other resonance (Supplementary Figure S2). Thus, stretching PDMS can tune the transmission spectra because coupling is the main driver for spectral tuning.

### 3.2 Reducing the Rayleigh Anomaly diffraction

**Figure 2:**
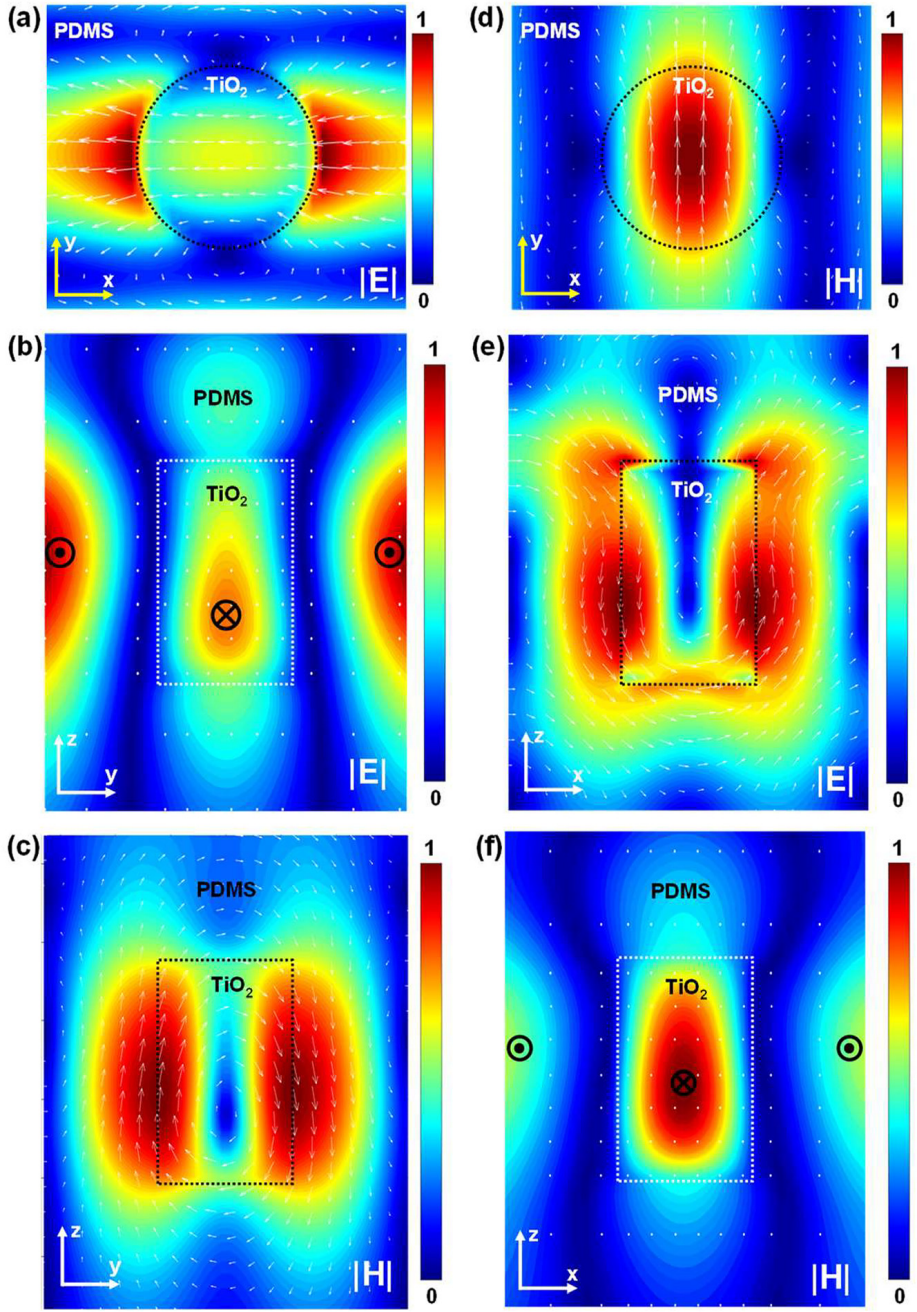
Electric and magnetic field intensity plotted along the vertical TiO_2_ nanodisc: (a)–(c) electric dipole resonance at λ = 502 nm and (d)–(f) magnetic dipole resonance at λ = 495 nm for the completely embedded TiO_2_ nanodisc array with a pitch of 330 nm.

To elucidate the origin of the electromagnetic resonances A and B in Figure 1(d), FDTD simulations were used to obtain the electric and magnetic field distributions within the nanodiscs. For example, the field distributions for nanodiscs with *P* = 330 nm are shown in Figure 2(a)–(c). For resonance A, the electric field aligned along the direction of incident polarization (Figure 2(a) and (b)), whereas the magnetic field vector curls in the cross section (*Y*–*Z* plane) shown in Figure 2(c). For resonance B, the electric field vector curls in the cross section (*X*–*Z* plane) shown in Figure 2(e), and the magnetic field vector was perpendicular to the direction of incident polarization (Figure 2(d) and (f)). Therefore, A is the electric dipole resonance and B is the magnetic dipole resonance. Notably, when the resonances shifted with decreasing spacing, crossover of the electric dipole resonance was observed at shorter wavelengths than that of the magnetic dipole resonance (Supplementary Figure S3). In Figure 1(d), the Rayleigh anomaly (RA) diffraction was observed close to the Mie-lattice resonances [22, 40], [41], [42]. This diffraction originates from the periodic structure that serves as a thin transmission grating and diffracts the impinging light [43]. To utilize the Mie-lattice resonances for practical applications, it is essential to reduce the RA intensity. Therefore, we added another parameter in the design of the array by creating an optically asymmetric structure which affects the RA.

The general condition for the RA diffraction was described by Strutt [40].

(1) $$n_{\text{sub}}Sin\left(\beta_{m}\right)=n_{\text{sup}}Sin\left(\alpha_{\text{inc}}\right)\pm m\lambda /P$$

where *n*_sub_ is the refractive index of the substrate, *β*_*m*_ is the diffraction angle in the substrate, *n*_sup_ is the refractive index of the superstrate, *a*_inc_ is the light angle in the superstrate, integer *m* is the order of the diffracted wave, *λ* is the wavelength of the light, and *P* is the pitch of the diffracting elements (the TiO_2_ nanodiscs). Because the incident light is normal to the nanodisc array (along the *z* axis), *a*_inc_ is 0° and *n*_sub_ and *n*_sup_ are identical. When the angle *β*_m_ is 90°, the RA occurs due to the propagation of the diffracted wave parallel to the grating. Thus, for the geometry of our system, the first order diffraction grating Equation (1) becomes:

(2) $$\lambda =n_{\text{sub}}\times P,$$

**Figure 3:**
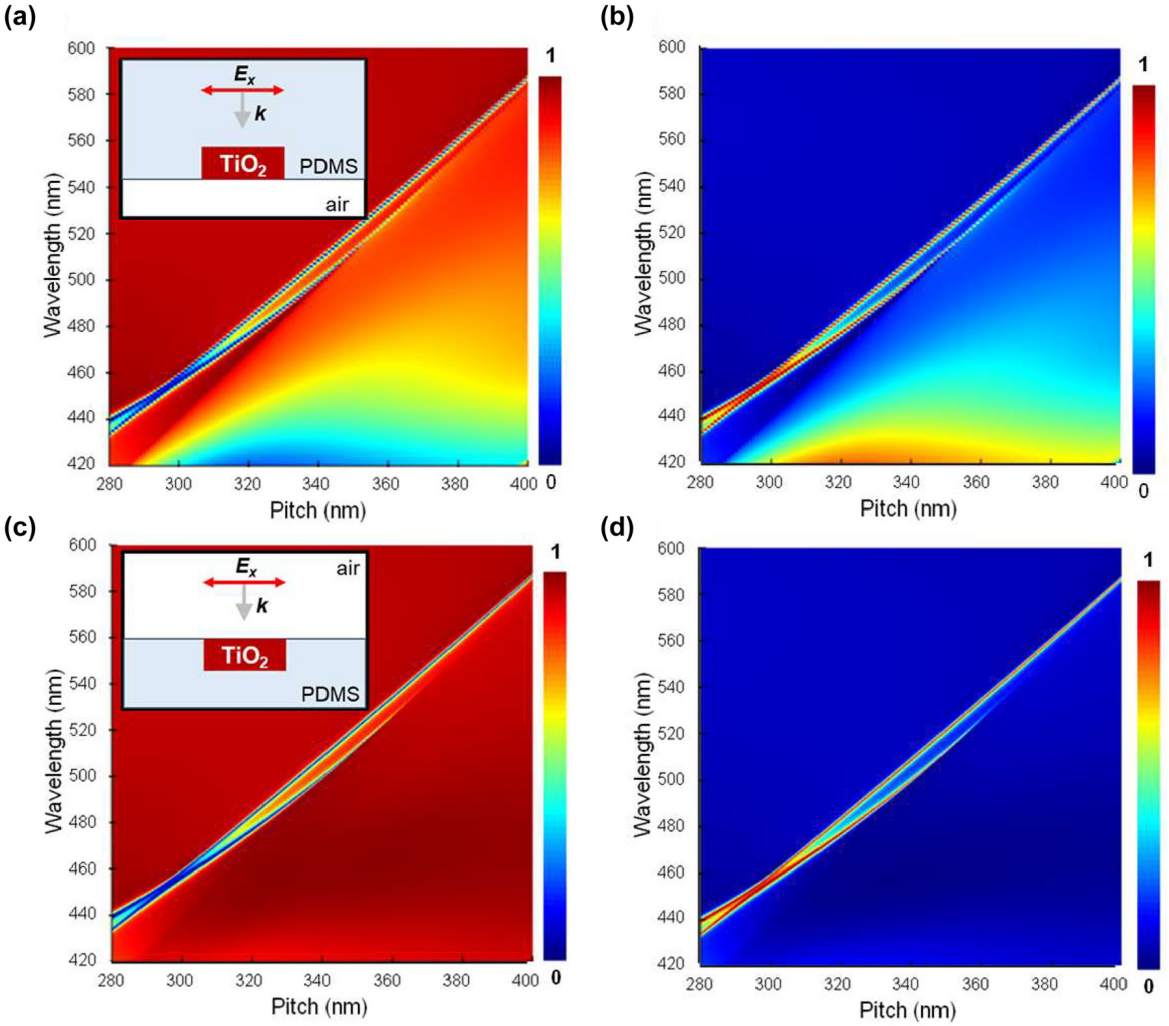
Asymmetric media structure of partially embedded TiO_2_ nanodiscs in PDMS with a diameter of 120 nm and thickness of 275 nm for Rayleigh anomaly resonance. (a) Transmittance and (b) reflectance spectra versus pitch of the TiO_2_ nanodiscs with light injected from the PDMS side and transmitted through air. (c) Transmittance and (d) reflectance spectra versus pitch of the TiO_2_ nanodiscs with light injected from air and transmitted through PDMS. Insets of panel (c) and (d) show the schematics of simulated TiO_2_ nanodisc structures.

Equation (2) indicates that changing the optical index of the embedding medium shifts the spectral location of the RA resonance. For example, reducing the refractive index of the medium caused a blueshift in the RA resonance (Supplementary Figure S4), but the change in the index also shifts the dipole resonances. Subsequently, a metasurface with shifted RA diffraction was designed, which consists of an optically asymmetric structure in which the top and bottom surfaces of the TiO_2_ nanodiscs are in contact with two media with different refractive indices (for example, PDMS and air). This structure was realized using partially embedded nanodiscs in PDMS. Figure 3(a) and (b) shows transmission and reflection when light was injected from the PDMS side, exhibiting a strong RA reflection. Figure 3(c) and (d) were obtained when light was injected from the air side, exhibiting a weak RA reflection. These results are attributed to the change in index at the interfaces. Because the effective index for the TiO_2_-PDMS composite is approximately 1.6 and that of air is 1, when light is injected from the PDMS side and propagates through air, the index is significantly reduced (Δ*n* = 0.6), which produces a strong reflection at the interface between the TiO_2_-PDMS composite and air. Conversely, when light is injected from the air side, a smaller decrease in the index was observed Δ*n* = 1.6–1.46 (the index of SiO_2_) = 0.14, causing a weaker RA reflection.

### 3.3 Fabrication of dynamically tunable TiO_2_ metasurface

**Figure 4:**
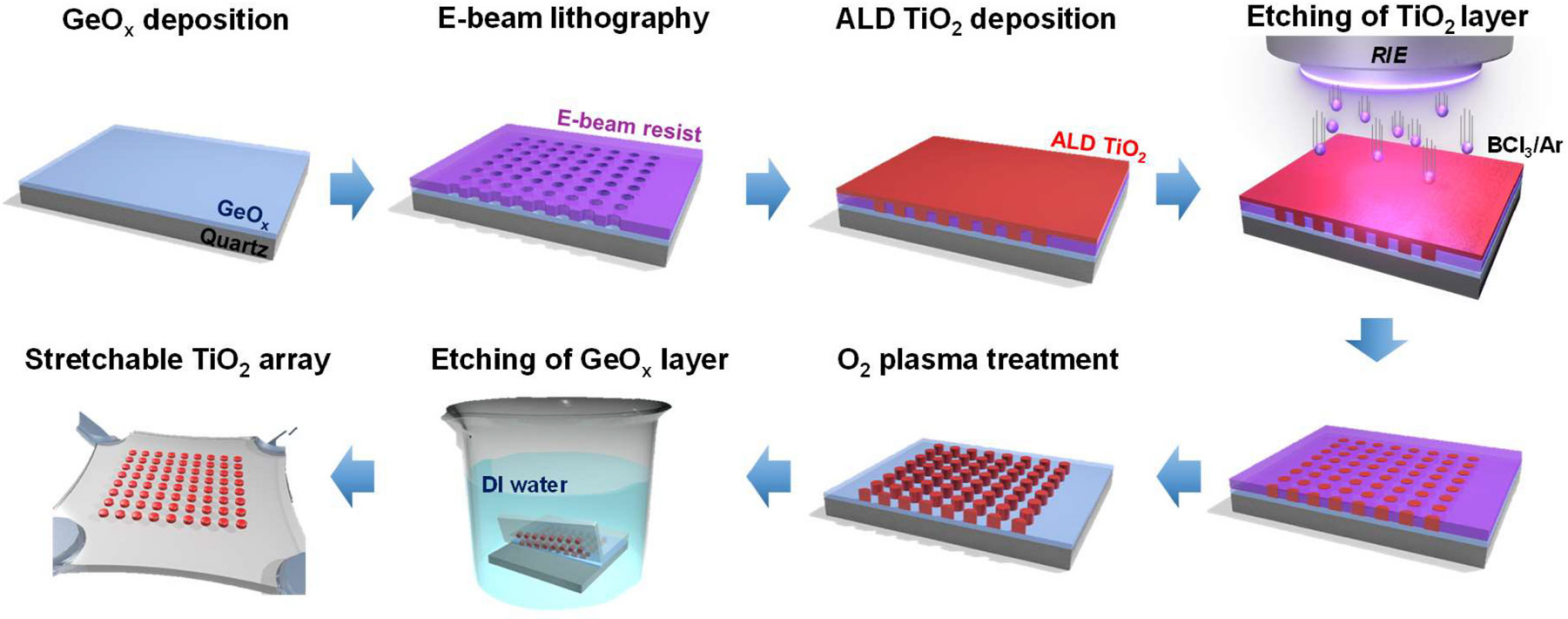
Fabrication process of PDMS-embedded TiO_2_ nanodisc array-based color filters.

The dynamic tuning of the TiO_2_ nanodisc color filtering system was realized experimentally by mechanically stretching PDMS with the partially embedded TiO_2_ nanodiscs. Stamping methods had been used to transfer nanostructures into flexible substrates [44, 45]. These transfers may form defects in the nanopattern, which reduces device fidelity. As shown in Figure 4, a different fabrication method for embedding TiO_2_ nanodiscs in PDMS was designed to produce defect-free structures. A sacrificial layer of GeO_*x*_ film, which can be dissolved in water, was deposited onto a glass substrate. This layer was coated with an electron-beam resist film and patterned by electron-beam lithography to produce a nanohole array. Subsequently, the nanoholes were filled with TiO_2_ using ALD, which has excellent step coverage. Near-perfect coverage was confirmed by evaluating a filled nano-trench (Figure S5). As expected, filling the nanoholes resulted in a thin TiO_2_ film on the electron-beam resist, which was completely removed by reactive ion etching. Thus, the height of TiO_2_ nanodiscs was determined by the electron-beam resist thickness. Therefore, the electron-beam resist was selectively etched by O_2_ plasma treatment to form a TiO_2_ nanodisc array on the GeOx layer. The use of a dry lift-off step provided a defect-free TiO_2_ metasurface. Subsequently, a PDMS solution was poured onto the TiO_2_ nanodiscs and cured. Finally, the device was immersed in water to remove the GeO_*x*_ layer. The combination of the lift-off step and sacrificial layer produces a defect-free stretchable metasurface. The scanning electron microscopy (SEM) image of as-fabricated TiO_2_ nanodiscs is shown in Figure S6.

### 3.4 Experimental demonstration of magnetic dipole resonances

**Figure 5:**
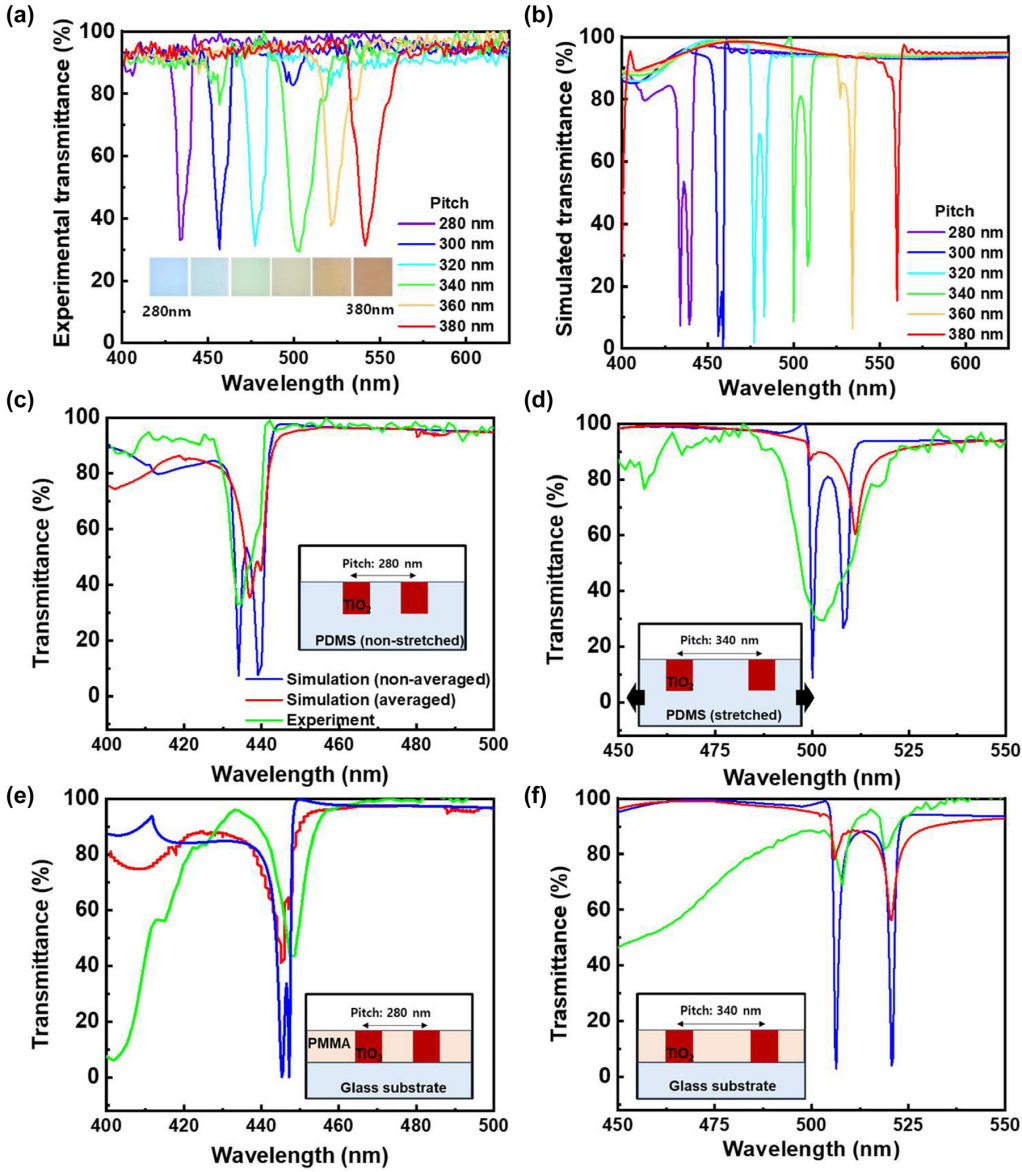
Dynamic color tuning by stretching PDMS-embedded TiO_2_ nanodiscs: (a) experimental and (b) simulated transmittance spectra of PDMS-embedded TiO_2_ nanodiscs with different pitches from 280 to 380 nm. The Insets in panel (a) show the optical microscopy images of PDMS-embedded TiO_2_ nanodiscs with different pitches from 280 to 380 nm. Transmittance spectra (c) and (d) for PDMS-embedded TiO_2_ nanodiscs and (e) and (f) for PMMA-coated TiO_2_ nanodiscs on glass substrate at a pitch of 280 and 340 nm – non-averaged simulated spectrum (blue line), simulated spectrum from averaged spectra with various angles of incident light (red line), and experimental spectrum (green line). The insets in panel (c)–(f) show the schematics of the TiO_2_ nanodiscs with different media.

The transmittance spectra of the metasurface were measured using a commercial hyperspectral imaging system. By mechanically stretching the PDMS, the pitch between the TiO_2_ nanodiscs was dynamically changed from 280 to 380 nm. Figure 5(a) shows spectra redshift with increasing pitch similar to the simulation results in Figure 1(d). For comparison, Figure 5(b) shows the simulated spectra of the metasurface dimensions, which corresponds with the experimental results, exhibiting a similar redshift trend. However, the simulated bands were much narrower with transmission minima of <10 % than the experiment ones, which exhibited broader transmission bands with minima of >30 %. Additionally, a single dip was observed in the experimental spectra, whereas the simulated spectra showed double dips representing the electric and magnetic dipole resonances. These differences are commonly reported for dielectric metasurface arrays [29, 30, 35] but the fact that often only one broad dip is observed remains unresolved. It is crucial to understand the origin of these differences to achieve the theoretical near-zero transmissions and be able to obtain high-quality resonances.

We suggest two points to investigate: (1) the origin of the differences between the computer simulations and experimental setups, and (2) the effect of the presence of slight variations in the array pitch and also the possibility of surface non-uniformity caused by the PDMS transfer or handling. For the former, an ideal case with a plane wave injection at a normal incidence was used in the simulations, whereas a light injection angle set by the numerical aperture (NA) of the objective lens was used experimentally. The experimental objective lens had NA = 0.13, which corresponds to a half angle of incidence equivalent to ±7.4° relative to the normal incidence. It is recognized that the transmission spectra of periodic dielectric nanostructures are dependent on the incidence angle [17, 24]. When the transmission spectra are averaged over the incidence angle, the resonance band broadening is reasonable. This was investigated by performing FDTD simulations in the incident angle range of 82.6°–97.4° with 0.3° steps. The simulated spectra with different incident angles are shown in Figure S7. A shift in the resonance position was observed with changes in polar angle (*θ*) and azimuthal angle (*φ*). The simulated data were averaged and plotted (in red) in Figure 5(c) with the transmission spectrum of the ideal case (plotted in blue) at a normal incidence for PDMS-embedded TiO_2_ nanodiscs with *P* = 280 nm. The averaged spectra exhibited a much less transmission minimum (38 %) than that at a normal incidence which were about 8 %. Notably, the averaged spectra exhibited overlapping spectra dips, which correspond with the experimental spectrum (Figure 5(c), plotted in green). A similar spectrum was obtained at *P* = 340 nm as shown in Figure 5(d). The transmission minimum is reduced for the angle averaged simulated plots compared with the normal incidence simulated plots, as shown in Figure 5(c) and (d). The weaker spectra dips in Figure 5(a) are due to the NA (0.13) of the objective lens in experiment compared with the normal incidence simulation in Figure 5(b).

The second hypothesis, which attributes the differences between experimental and simulated results to slight variations in the spatial dimensions during device fabrication was also investigated. Spatial variations and surface non-uniformity can occur during the (a) transfer of the TiO_2_ metasurface to PDMS or (b) mounting of the thin flexible metasurface onto the mechanical stretching mount. Furthermore, these non-uniformities can be amplified by the stretching process. A small local variation in the position of the array elements may cause incoherency in strongly coupled modes, resulting in broader collective resonance. To investigate this hypothesis, TiO_2_ nanodiscs were formed on a rigid glass substrate to eliminate bending, twisting, or variations in the pitch between the nanodiscs during the transfer process. A structure similar to PDMS-embedded TiO_2_ metasurface was implemented by fabricating the TiO_2_ metasurface on a glass substrate and embedding the metasurface in a polymethyl methacrylate (PMMA) acrylic glass with a thickness equal to the nanodisc height. The refractive index of PMMA is approximately 1.48, which is close to that of PDMS (*n* = 1.46), and thus the two systems are similar. The low viscosity of the PMMA solution enabled easy filling between the TiO_2_ nanodiscs by spin coating. The dipole resonances of the PMMA-containing structure were simulated at a normal incidence and incident light with different angles. Figure 5(e) shows the simulated transmittance spectrum at a normal incidence (blue) and averaged transmittance spectra with different angles (red) for the PMMA-coated TiO_2_ metasurface on the glass substrate (*P* = 280 nm). The averaged spectra of the glass/PMMA metasurface exhibited a similar trend with overlapping bands. It was experimentally confirmed that the spectra (green in Figure 5(e)) for the glass/PMMA metasurface at *P* = 280 nm resembles the simulation spectra obtained with illumination angle averaged (red in Figure 5(e)) for this geometry. Notably, for the glass/PMMA metasurface (green in Figure 5(e)), the double transmission dips were experimentally observed and resemble those in the simulation. In addition, the measured dip was sharper with a full width at half maximum (FWHM) of 7.2 nm, compared to the case of metasurface embedded in PDMS (green in Figure 5(c)) with FWHM = 9.0 nm. Subsequently, as shown in Figure 5(f), the pitch was increased to 340 nm, and the experimental data (in green) for the electric and magnetic dipole resonances show two distinct dips which are similar to the case for the normal incidence light in the averaged simulated spectra. Based on these results, we concluded that the illumination angle variations strongly influence the intensities of the transmission dips. Moreover, the spatial non-uniformity induced in the metasurface during the mechanical stretching causes band broadening due to pitch variations. The measured FWHM of the spectra of the glass/PMMA metasurface is 3.4 nm (green in Figure 5(f)) was larger than that of the averaged simulation (red in Figure 5(f)), which has FWHM = 2.1 nm.

According to Figure 5(c)–(f), we concluded that pitch variation during mechanically stretching of the PDMS is likely more dominant for broadening the experimental spectra. However, these variations do not negatively affect the functionality of the metasurface as a narrow-band tunable filter for the dynamic selection of visible wavelengths. To accurately control the pitch of the PDMS-embedded TiO_2_ nanodiscs, a stretching system is being developed using dielectric elastomer actuators [46] instead of a mechanical force. Using the dielectric elastomer actuators, it is possible to stretch along the radial direction and control the stretching electrically. Thus, a more uniform and accurate control of nanodisc array pitch is expected, which may produce narrow-band TiO_2_ nanodisc color filters.

## 4 Conclusions

This study presents a reliable method for fabricating dynamically tuned TiO_2_-based dielectric metasurfaces using a mechanically stretchable elastomer. Dynamically tunable color filters were demonstrated by mechanically stretching the PDMS-embedded dielectric metasurface up to 43 % and regulating the spectral location of the Mie lattice resonances by changing the pitch of the array. Moreover, sharp, and variable wavelength spectra transmission dips in the range of 435–580 nm were achieved. Additionally, the Rayleigh anomaly diffraction was shifted by embedding TiO_2_ nanostructures in an optically asymmetric structure enabling a more flexible engineering of transmission filters.

From simulations and experiments we showed that the non-uniformity of the stretching can increase the broadness of the lattice resonances, while focusing the incident light through large NA optics leads to averaging the transmittance spectra and reduces the quality factor.

Due to the high transparency of TiO_2_ and the PDMS in the visible region, the PDMS-embedded TiO_2_ dielectric metasurfaces provide lossless resonances. With a process compatible with the industrial CMOS fabrication process, we created defect-free photonic dielectric metasurfaces that can be used as narrow band filters in the visible light. The tuning region of the developed device coincides with the spectral region of visible light cameras. Thus, by coupling these filters with appropriate photodetectors, they can be used in hyperspectral imaging and other sensing applications.

## Supplementary Material

Supplementary Material Details
