# Supplementary material for: Dynamic selection of visible wavelengths using resonant TiO2 nanostructures
Source: Nanophotonics. 2023 May 3;12(11):1995–2005. doi: 10.1515/nanoph-2023-0057 (PMC11501797; doi:10.1515/nanoph-2023-0057)
Supplement: Supplementary file 1 — Supplementary Material Details [file j_nanoph-2023-0057_suppl_001.doc]

**Supplementary Material**

Han-Don Um, Deokjae Choi, Amit Solanki, Emerald Huang, Kwanyong Seo, and Fawwaz Habbal*

Dynamic Selection of Visible Wavelengths Using Resonant TiO_2_ Nanostructures


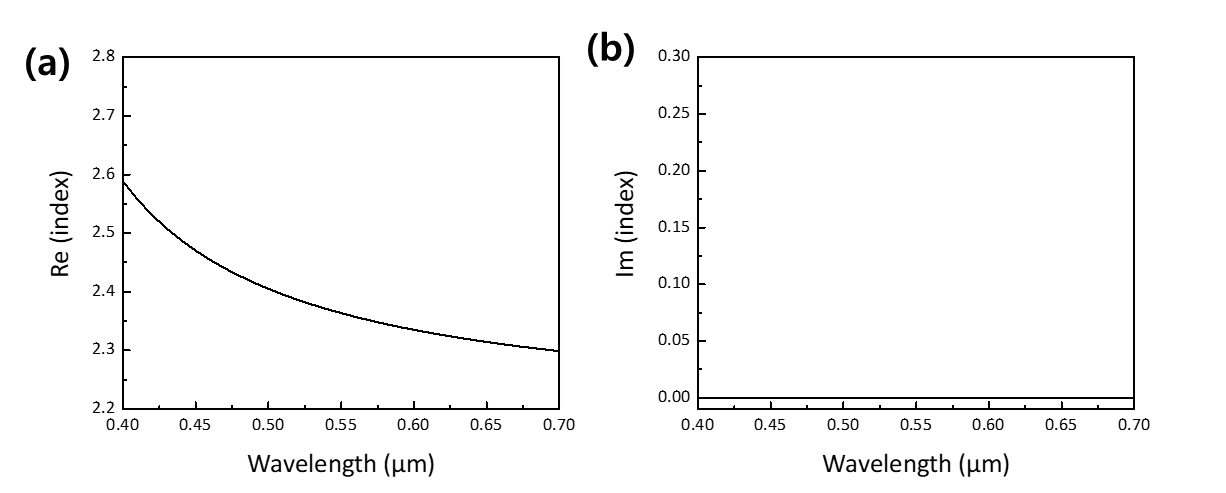


**Figure S1.**  Experimental refractive index (a) and extinction coefficient (b) of a TiO_2_ layer.

**Figure S2.** (a) Transmittance spectra versus different pitches of the fully embedded TiO_2_ nanodiscs with a diameter of 120 nm and a thickness of 275 nm. Inset of panel (a) is schematics of TiO_2_ nanodiscs embedded into PDMS. (b) Simulated transmittance spectra of fully embedded TiO_2_ nanodiscs with different pitches.

**Figure S3.** Field patterns of electric and magnetic dipole resonance. (a-c) Electric dipole resonance at λ = 439 nm and (d-f) magnetic dipole resonance at λ = 444 nm, for the TiO_2_ nanodisc array with a pitch of 280 nm.

**Figure S4.** Transmittances of TiO_2_ nanodiscs fully surrounded by homogeneous media with different indices, (a) 1.46, (b) 1.3, and (c) 1.1. Dashed green lines are plots according to simplified Rayleigh Anomaly equation.


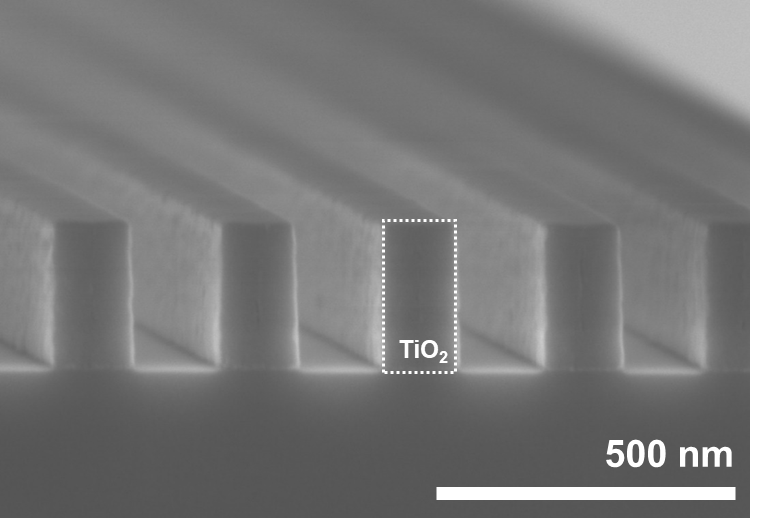


**Figure S5.** Cross-sectional scanning electron microcopy image of TiO_2_ nano-wall.

**Figure S6.** Scanning electron microcopy image of Pt-coated TiO_2_/PDMS metasurface where the TiO_2_ nanodiscs are highlighted in false green color.

**Figure S7.** Simulated transmission spectra of PDMS-embedded TiO_2_ nanodiscs with different polar (*θ*) and azimuthal angles (*φ*). (a) *φ* = 4.5 ° and *θ* = 0 ~ 85 °, (b) (a) *φ* = 0~ 7.2 ° and *θ* = 0 °.
